# The proportion of chronic periprosthetic joint infection patients with *Candida* isolates

**DOI:** 10.5194/jbji-11-31-2026

**Published:** 2026-01-13

**Authors:** Samuelson E. Osifo, Adrian Santana, Michael F. Shannon, Victoria R. Wong, Caroline F. Tyndall, Christian Cisneros, Niosha Parvizi, Brian A. Klatt, Johannes F. Plate, Nicolas S. Piuzzi, Kenneth L. Urish

**Affiliations:** 1 Department of Orthopaedic Surgery, University of Pittsburgh, Pittsburgh, PA 15213, USA; 2 University of Pittsburgh School of Medicine, Pittsburgh, PA 15213, USA; 3 Cleveland Clinic Foundation, Cleveland, OH 44195, USA; 4 Arthritis and Arthroplasty Design Group, The Bone and Joint Center, Magee-Womens Hospital of the University of Pittsburgh Medical Center, Pittsburgh, PA 15212, USA; 5 Department of Orthopaedic Surgery, Department of Bioengineering, and Clinical and Translational Science Institute, University of Pittsburgh, Pittsburgh, PA 15213, USA

## Abstract

**Introduction**: Fungal periprosthetic joint infection (PJI) has historically been reported in 1 %–2 % of cases, with *Candida* species accounting for most isolates. However, the true incidence is likely underestimated. Standard aerobic and anaerobic culture techniques have limited sensitivity for detecting fungi, single positive fungal cultures are often excluded or inconsistently classified, culture-negative infections may mask low-burden fungal pathogens, and polymicrobial cultures may obscure the contribution of fungal organisms. The objective of this study was to quantify the burden of potentially unrecognized fungal involvement and provide a more accurate estimate of the incidence of *Candida*-associated PJI. **Methods**: Following a systematic literature search, we performed a quantitative sensitivity analysis using imputation with informative missingness odds ratios (IMORs). Reported *Candida* cases were adjusted for four predefined sources of under-ascertainment: single positive cultures, negative cultures, polymicrobial cultures, and variability in fungal culture sensitivity. **Results**: 23 studies met inclusion criteria, reporting a total of 28 253 PJI patients, of whom 590 had *Candida* involvement (2.1 %; range 0.9 %–10.1 %). After imputation for missing data, the estimated proportion of PJI cases involving *Candida* ranged from 1.4 %–13.6 %, with a mean of 5.1 %. The odds ratios for known risk factors for chronic refractory PJI exceeded 2.0, suggesting the proportion of *Candida* in this population likely exceeds 10 %. **Conclusion**: The involvement of *Candida* in PJI is likely underreported. The adjusted incidence is approximately 5 % across all PJI cases. Among patients with chronic refractory PJI, especially those that have failed multiple surgeries, the incidence of *Candida* PJI is approximately 10 %. **Level of Evidence**: Level III.

## 1 Introduction

Periprosthetic joint infection (PJI) remains the most serious complication of hip and knee arthroplasty globally and the leading cause of early revision in total knee arthroplasty (Del Pozo and Patel, 2009; Kurtz et al., 2007). Fungal PJI has traditionally been considered rare, historically reported in 1 %–2 % of PJI cases (Sambri et al., 2022; Sidhu et al., 2019; Tande and Patel, 2014). However, contemporary evidence from large primary cohorts suggests that fungal organisms may be present in a substantially higher proportion (up to 10 %) of PJI cases (Brown et al., 2018; Cao et al., 2025). *Candida* species account for approximately 95 % of culture-confirmed fungal PJIs (Benito et al., 2016; Koutserimpas et al., 2022; Herndon et al., 2023; McCulloch et al., 2023).

Several factors likely contribute to systematic underestimation of fungal involvement in chronic PJI. Conventional aerobic and anaerobic culture methods have reduced sensitivity for *Candida*, particularly in low-burden infections or polymicrobial communities, making both single isolates and mixed fungal–bacterial infections difficult to detect (Watanabe et al., 2024). Underreporting may also occur in culture-negative cases, especially in institutions without optimized fungal culture protocols or prolonged incubation. Diagnostic uncertainty is further compounded when a single *Candida* isolate is dismissed as contamination, leading to misclassification. Additionally, many published series further aggregate acute and chronic PJI, although fungal infections occur predominantly in chronic refractory cases.

The objective of this study was to assess how these diagnostic and reporting limitations influenced the observed incidence of fungal PJI. Specifically, we aimed to (1) establish the baseline proportion of *Candida* isolates among confirmed PJI cases; (2) identify the frequency of missing, excluded, or misclassified variables that could obscure true fungal involvement; and (3) quantify the adjusted incidence of *Candida*-associated PJI after correcting for potential underreporting.

## 2 Methods

### 2.1 Study design

This study was a quantitative sensitivity analysis of published clinical series reporting microbiologic findings in confirmed periprosthetic joint infection (PJI). The primary outcome was the proportion of PJI cases with evidence of *Candida* involvement. The analysis was designed to evaluate the impact of incomplete or inconsistently reported microbiologic data on observed estimates of *Candida* involvement across heterogeneous clinical studies.

### 2.2 Data sources and study selection

**Figure 1:**
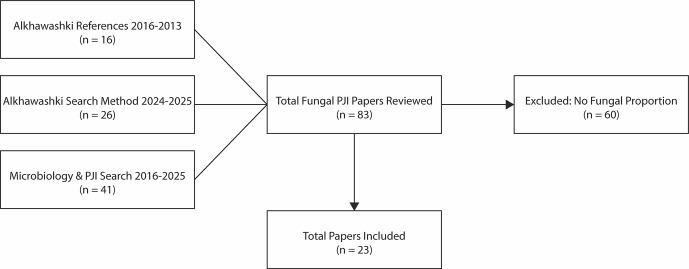
Flow diagram of literature search and study selection.

A recent meta-analysis identified English-language publications reporting outcomes on ≥5 patients with diagnosed hip or knee fungal PJI between 2009 and 2023 (Alkhawashki et al., 2025). All studies published between 2016 and 2023 included in this meta-analysis were reviewed. To extend capture beyond this cohort, a structured literature search was independently conducted for studies published from January 2024 through October 2025 using PubMed. The primary search string was “fungal periprosthetic joint infection”, applied to titles and abstracts. In parallel, a secondary PubMed search was performed, following the methodology described by Tai et al. (2022a), using the Boolean search strategy “hip” OR “knee” AND “periprosthetic joint infection” AND “microbiology aetiology” for publications from 2016 through October 2025. Reviewed studies were included if they reported microbiologic results from preoperative or intraoperative samples and allowed calculation of the proportion of PJI cases with evidence of *Candida* involvement. Studies were excluded if microbiologic data were insufficient to determine *Candida* involvement. This process yielded 23 studies for inclusion (Fig. 1).

### 2.3 Identification of missing microbiologic data

The proportion of PJI patients with *Candida*-positive preoperative or intraoperative cultures was calculated for each study included. Review of included studies revealed inconsistent methodology and reporting practices, resulting in missing diagnostic data relevant to the calculated proportion of *Candida* involvement. Four prespecified sources of missingness were identified:

1. Culture-negative PJI cases included without clarification of fungal assessment;
2. Polymicrobial PJI cases without species-level reporting, where fungal organisms may have been present but not specified;
3. Exclusion of single positive *Candida* isolates, commonly dismissed as contaminants;
4. Variable sensitivity of microbiologic methods, with potential under-detection of fungal organisms.

### 2.4 Sensitivity analysis and imputation strategy

A quantitative sensitivity analysis was conducted using imputation with informative missingness odds ratios (IMORs) for each missingness category, following methods for imputing missing outcome data in meta-analyses of clinical trials (Higgins et al., 2008). For each study, the expected number of missed *Candida* cases was estimated under both best-case and worst-case scenarios. These expected cases were added to the reported number of *Candida* infections, and minimum and maximum adjusted proportions were calculated. A sample size-weighted mean and range of expected *Candida* proportions was generated across all included studies.

Consistent with Higgins et al. (2008), the analysis did not assume that missing patients were either entirely “all *Candida*” or “no *Candida*.” Instead, each category of missingness was assigned a relative risk (RR) of *Candida* involvement based on published clinical data. Applying these literature-derived probabilities provides more realistic estimates and reduces the standard error compared with traditional all-or-none assumptions. The additional number of expected *Candida* cases for each missingness category was calculated as $C_{i}={\mathrm{RR}}_{i}\times m_{i}$ , where $m_{i}$ represents the number of PJI cases affected by that specific source of missingness.

The number of PJI patients with missing data for *Candida* is often reported directly in the paper (e.g., number of culture-negative patients). If such patients are excluded, or not reported and assumed to be excluded in worst-case sensitivity calculations, the number of missing patients ( $m_{i}$ ) was calculated by multiplying the total number of PJI patients in the study ( $N_{i}$ ) by the proportion ( $p_{i}$ ) of PJI patients in the literature with the same reason for missingness (e.g., percentage of culture-negative PJI cases): $m_{i}=p_{i}\times N_{i}$ .

The relative risk of *Candida* for each missingness category (RR_*i*_) was derived from the literature. For missed cases attributable to insensitive microbiologic test methods, RR was calculated as the difference in the probability of detecting *Candida* using high-sensitivity methods compared with conventional culture techniques reported in the publication. Imputation was restricted to *Candida* involvement because fungal organisms are inconsistently assessed and variably reported across published PJI series, with frequent exclusion of single positive cultures, lack of species-level reporting in polymicrobial infections, and heterogeneous use of high-sensitivity diagnostic methods. These factors introduce systematic uncertainty in estimates of fungal involvement that cannot be addressed by standard reporting alone.

### 2.5 Best-case and worst-case scenarios

Best-case and worst-case scenarios were defined to bound the plausible range of *Candida* involvement under varying assumptions regarding missing microbiologic data. The best-case and worst-case scenarios of the sensitivity analysis for each missingness reason were determined (Table 1). These scenarios depend on whether PJI patients were included in the study without microbial data, specifically excluded from the study, or not reported (and therefore potentially included or excluded). When patients with expected *Candida* involvement were known to be missing, they were added to both the best-case and worst-case scenarios. If the number of PJI patients affected by a missingness category was not reported, no imputation was performed for the best-case scenario (Add 0). When PJI patients were excluded or assumed to be excluded in worst-case scenarios, the number of missing PJI patients for that reason ( $N_{i}$ ) was added to the total number of PJI patients before recalculating the proportion of patients expected to have *Candida* involvement, to avoid overestimation.

**Table 1:** Imputation methods by missingness category.

	Reason for missingness			
How missingness was reported	Culture-negative cases	Polymicrobial cases (species not specified)	Single positive *Candida* isolate excluded	Absence of high-sensitivity microbiologic testing
Included in total PJI count and *Candida* count	No imputation	No imputation	No imputation	No imputation
Included in total PJI count but not in *Candida* count	Best – Add $C_{1}$ Worst – Add $C_{1}$	Best – Add $C_{2}$ Worst – Add $C_{2}$		
Excluded	Best – Add $C_{1}$ (Add $N_{1}$ to number of PJI) Worst – Add $C_{1}$ (Add $N_{1}$ to number of PJI)	Best – Add $C_{2}$ (Add $N_{2}$ to number of PJI) Worst – Add $C_{2}$ (Add $N_{2}$ to number of PJI)	Best – Add $C_{3}$ (Add $N_{3}$ to number of PJI) Worst – Add $C_{3}$ (Add $N_{3}$ to number of PJI)	Best – Add $C_{4}$ Worst – Add $C_{4}$
Not reported	Best – Add $C_{1}$ Worst – Add $C_{1}$ (Add $N_{1}$ to number of PJI)	Best – Add 0 Worst – Add $C_{2}$ (Add $N_{2}$ to number of PJI)	Best – Add 0 Worst – Add $C_{3}$ (Add $N_{3}$ to number of PJI)	Best – Add 0 Worst – Add $C_{4}$

PJI – periprosthetic joint infection.

### 2.6 Informative missingness odds ratios (IMORs)

The relative risks associated with each missingness category were derived from published literature reporting the proportion of cases with *Candida* isolates associated with each source of missingness, including the following:

1. Detection of *Candida* in culture-negative PJI using next-generation sequencing (NGS).
2. The proportion of *Candida* in polymicrobial PJI.
3. The frequency of single positive *Candida* cultures in PJI.
4. The detection of *Candida* by high-sensitivity microbial assays missed by conventional cultures.

The IMOR for each category was calculated as a weighted average across included studies (Table 2).

**Table 2:** Informative missingness odds ratios (IMORs) for each missingness category.

Reference	Number	Candida	Candida	Rate of	High-
	of PJI	rate in	rate in	single	sensitivity
	cases	culture-	polymicrobial	*Candida*	test method
		negative	PJI	cultures	
		PJI		in PJI	
Brown et al. (2018)	3525			0.37	
Tai et al. (2022a)	2,067			0.49	
Tai et al. (2022b)	1162				0.35
van den Bijllaardt et al. (2019)	90				0.30
Enz et al. (2021)	117		0.78		
Cao et al. (2025)	278		0.27		
Froschen et al. (2022)	432		0.05		
Mei et al. (2023)	91		0.14		
Ull et al. (2020)	124		0.17		
Goswami et al. (2022)	301	0.08			
Lin et al. (2025)	232	0.13			
Weighted average		0.10	0.21	0.41	0.35

PJI – periprosthetic joint infection.

## 3 Results

A total of 23 studies met inclusion criteria, reporting a combined total of 28 253 PJI patients (Table 3). *Candida* PJI was identified in 533 patients (1.9 %). When patients with a single positive *Candida* culture were included, the total increased to 590 patients (2.1 %). Across all included studies, the reported incidence of *Candida* PJI ranged from 0.9 % to 10.1 %.

**Table 3:** Reported proportion of confirmed PJI cases with a *Candida* isolate.

Reference	Number	Number of	Single	Total	*Candida*
	of PJI	*Candida*	cultures	cases	proportion
	cases	cases	excluded	with	
	reported	reported		*Candida*	
				isolates	
Baecker et al. (2021)	623	20		20	3.2 %
Lee et al. (2024)	181	13		13	7.2 %
Lin et al. (2025)	271	16		16	5.9 %
Lyu et al. (2024)	219	13		13	5.9 %
Morreel et al. (2025)	187	2		2	1.1 %
Tai et al. (2022b)	1162	40	39	79	3.4 %
Wu et al. (2025)	201	4		4	2.0 %
Aunon et al. (2025)	499	12		12	2.4 %
Froschen et al. (2022)	432	17		17	3.9 %
Jiang et al. (2025)	255	7		7	2.7 %
Ull et al. (2020)	124	5		5	4.0 %
Brown et al. (2018)	3525	31	18	49	0.9 %
Herndon et al. (2023)	3989	73		73	1.8 %
Tsai et al. (2019)	294	10		10	3.4 %
Budin et al. (2025)	3645	47		47	1.3 %
Cao et al. (2025)	278	28		28	10.1 %
Kuo et al. (2018)	1184	29		29	2.4 %
Sidhu et al. (2019)	1189	22		22	1.9 %
Benito et al. (2016)	2524	30		30	1.2 %
Ergin et al. (2024)	2569	49		49	2.0 %
Ergin et al. (2025)	4261	45		45	1.1 %
Haraldsdottir et al. (2025)	293	6		6	2.0 %
Yang et al. (2024)	348	14		14	4.0 %
Totals and grand mean (%)	28 253	533	57	590	2.1 %

Each study was assessed for missing diagnostic data that could lead to underestimation of *Candida* involvement (Table 4). The major sources of missingness included (1) culture-negative PJI cases in which fungal involvement could not be excluded, (2) polymicrobial PJI cases where the full microbial profile was not reported, (3) exclusion or non-classification of single positive *Candida* isolates, and (4) the absence of high-sensitivity microbiologic testing such as fungal cultures, next-generation sequencing (NGS), or sonication.

**Table 4:** Summary of missing data categories requiring imputation for each study.

	Imputation for missing data			
Reference	Culture-	Polymicrobial	Excluded	Absence
	negative	PJI cases	single	of high-
	PJI cases	without	positive	sensitivity
		species	*Candida*	microbiologic
		specification	isolates	testing
Missing data for one reason				
Baecker et al. (2021)	Yes	n/a	n/a	n/a
Lee et al. (2024)	Yes	n/a	n/a	n/a
Lin et al. (2025)	Yes	n/a	n/a	n/a
Lyu et al. (2024)	n/a	n/a	n/a	Yes
Morreel et al. (2025)	Yes	n/a	n/a	n/a
Tai et al. (2022b)	Yes	n/a	n/a	n/a
Missing data for two reasons				
Wu et al. (2025)	Yes	Yes	n/a	n/a
Aunon et al. (2025)	Yes	n/a	Yes	n/a
Froschen et al. (2022)	Yes	n/a	Yes	n/a
Jiang et al. (2025)	Yes	n/a	Yes	n/a
Ull et al. (2020)	Yes	n/a	Yes	n/a
Brown et al. (2018)	Yes	n/a	n/a	Yes
Herndon et al. (2023)	Yes	n/a	n/a	Yes
Missing data for three reasons				
Tsai et al. (2019)	Yes	n/a	Yes	Yes
Budin et al. (2025)	Yes	n/a	Yes	Yes
Cao et al. (2025)	Yes	n/a	Yes	Yes
Kuo et al. (2018)	Yes	n/a	Yes	Yes
Sidhu et al. (2019)	Yes	n/a	Yes	Yes
Missing data for four reasons				
Benito et al. (2016)	Yes	Yes	Yes	Yes
Ergin et al. (2024)	Yes	Yes	Yes	Yes
Ergin et al. (2025)	Yes	Yes	Yes	Yes
Haraldsdottir et al. (2025)	Yes	Yes	Yes	Yes
Yang et al. (2024)	Yes	Yes	Yes	Yes

n/a = not applicable; no imputation required for that category.

Among all included studies, only the series by Lyu et al. (2024), which used NGS to detect *Candida* in culture-negative cases, did not require imputation for the culture-negative category. Missingness due to unreported polymicrobial species required imputation in 6 studies, exclusion of single positive *Candida* isolates in 13 studies, and absence of high-sensitivity culture methods in 12 studies. The incidence of *Candida* PJI was then recalculated after applying imputation for each missingness category (Table 5).

**Table 5:** Sensitivity analysis: minimum and maximum expected proportion of *Candida* in confirmed PJI cases.

Reference	Total	Reported	Reported	Minimum	Maximum
	PJI	*Candida*	*Candida*	expected	expected
	cases	cases	proportion	*Candida*	*Candida*
				proportion	proportion
Baecker et al. (2021)	623	20	3.2 %	3.2 %	4.0 %
Lee et al. (2024)	181	13	7.2 %	9.4 %	9.4 %
Lin et al. (2025)	271	16	5.9 %	6.3 %	6.3 %
Lyu et al. (2024)	219	13	5.9 %	5.9 %	6.4 %
Morreel et al. (2025)	187	2	1.1 %	1.9 %	1.9 %
Tai et al. (2022b)	1162	79	3.4 %	7.3 %	7.3 %
Wu et al. (2025)	201	4	2.0 %	5.0 %	5.0 %
Aunon et al. (2025)	499	12	2.4 %	2.4 %	3.9 %
Froschen et al. (2022)	432	17	3.9 %	3.9 %	5.1 %
Jiang, et al. (2025)	255	7	2.7 %	3.9 %	4.7 %
Ull et al. (2020)	124	5	4.0 %	4.8 %	4.8 %
Brown et al. (2018)	3525	49	0.9 %	1.4 %	2.9 %
Herndon et al. (2023)	3989	73	1.8 %	2.7 %	3.3 %
Tsai et al. (2019)	294	10	3.4 %	6.1 %	6.8 %
Budin et al. (2025)	3645	47	1.3 %	2.2 %	3.5 %
Cao et al. (2025)	278	28	10.1 %	12.2 %	13.6 %
Kuo et al. (2018)	1184	29	2.4 %	3.2 %	4.6 %
Sidhu et al. (2019)	1189	22	1.9 %	1.9 %	4.0 %
Benito et al. (2016)	2524	30	1.2 %	5.5 %	6.9 %
Ergin et al. (2024)	2569	49	2.0 %	8.7 %	9.8 %
Ergin et al. (2025)	4261	45	1.1 %	7.1 %	8.3 %
Haraldsdottir et al. (2025)	293	6	2.0 %	5.8 %	7.1 %
Yang et al. (2024)	348	14	4.0 %	10.3 %	11.7 %
Total and grand mean (%)	28 253	590	2.1 %	5.1 %	

PJI – periprosthetic joint infection.

After adjusting for missing data, the expected proportion of *Candida* PJI increased to 5.1 % across all studies (range: 1.4 %–13.6 %). This estimate was derived by adding the expected number of additional *Candida* cases for each missingness variable (Table A1). Most adjustments were attributable to unreported microbial species in polymicrobial cases (53 %) and unknown microbiology in culture-negative PJI cases (30 %).

As a sensitivity check, the subset of six studies requiring only one imputation correction, representing the lowest potential risk of error from multiple imputations, yielded an expected *Candida* incidence of 5.9 % (range: 1.9 % to 9.4 %). This is consistent with the overall adjusted estimate and supports the robustness of the findings.

## 4 Discussion

This quantitative sensitivity analysis suggests that the contribution of *Candida* to PJI is substantially underestimated in the published literature. After imputing missing microbiologic data across 28 253 confirmed PJI cases from 23 clinical studies, the expected proportion of *Candida*-associated PJI increased to approximately 5 %–6 % (range: 1.4 %–13.6 %). This estimate is more than double the conventionally reported incidence of 1 %–2 % and represents a clinically meaningful difference for diagnosis, treatment planning, and prognostication. By explicitly accounting for multiple, well-described sources of microbiologic under-ascertainment, this analysis provides an adjusted estimate of *Candida* involvement that complements existing epidemiologic reports. Notably, more than half of the included studies were published within the past decade, with a marked increase in reports from 2024–2025, reflecting growing recognition of fungal involvement in PJI.

Missing data were common and required imputation for all of the 23 studies. The most frequent source of missingness was failure to report organism-level microbiology in polymicrobial infections. Because polymicrobial PJI appears to carry a relatively high expected proportion of *Candida*, greater than 20 % in this analysis, failure to characterize all identified organisms may substantially underestimate fungal involvement.

Although the expected proportion of *Candida* was calculated for all reported PJI cases, fungal pathogens are most frequently encountered in chronic refractory infections and in sub-populations with risk factors associated with chronic PJI treatment failure. In a recent series of 193 chronic PJI cases treated with two-stage exchange, *Candida* accounted for 7.2 % of infections, increasing to 9.4 % when adjusted with the current methodology (Lee et al., 2024). Multiple studies have demonstrated strong associations between *Candida* PJI and clinical factors characteristic of chronic refractory infection, including prolonged infection duration, multiple prior surgeries, obesity, diabetes mellitus, presence of a draining sinus, polymicrobial infection, and recent antibiotic exposure. The reported odds ratios range from 2.2 to 7.2 (Aunon et al., 2025; Ergin et al., 2024, 2025; Gross et al., 2021; Kuo et al., 2018; Luo et al., 2025; Riaz et al., 2020). These data collectively support an expected *Candida* proportion of approximately 10 % in chronic refractory PJI cases, the upper estimate of our quantitative analysis.

An important consideration is whether imputation of missing microbiologic data selectively inflates estimates of *Candida* involvement or instead reflects broader limitations in microbiologic characterization within published PJI series. Staphylococcal species, particularly *Staphylococcus aureus* and *S. epidermidis*, account for more than half of reported PJI cases and are more consistently identified and reported in routine clinical practice. However, even for bacterial pathogens, culture-negative and polymicrobial cases remain common, indicating that missingness reflects incomplete or heterogeneous microbiologic reporting rather than organism-specific bias. This analysis does not suggest that *Candida* replaces bacterial pathogens within culture-negative infections but rather that fungal involvement is under-recognized within a subset of these cases. Collectively, these findings underscore that missingness imputation does not “create” fungal infections but instead quantifies uncertainty arising from inconsistent application and reporting of microbiologic diagnostic methods.

This study has several limitations. The inability to stratify results by specific risk factors limits granularity and reflects a broader challenge in fungal PJI research: microbiologic data are inconsistently reported and often aggregated across heterogeneous patient populations. Additional limitations include reliance on retrospective studies, the relatively small cohort of confirmed fungal PJI cases, and the limited number of publications with sufficient detail to calculate risk ratios for each category of missing data. The imputed expected proportions represent extrapolations based on available information rather than true corrections derived from recovered data, and errors may be magnified in studies with multiple sources of missing data.

## 5 Conclusion

A quantitative sensitivity analysis of missing microbiologic data suggests that the contribution of *Candida* to PJI is likely underestimated. The expected proportion of *Candida*-associated PJI is at least 5 % of all confirmed PJI cases and closer to 10 % among patients with chronic refractory infections. The discrepancy between reported and expected incidence highlights the need for prospective, multi-center studies that incorporate high-sensitivity fungal diagnostics, including prolonged culture, molecular testing, and standardized reporting of polymicrobial infections. More accurate estimates of *Candida* involvement in PJI will strengthen risk stratification and better inform surgical and antimicrobial decision-making, particularly for patients with complex or treatment-resistant infection.

## Data Availability

No data sets were used in this article.

## Appendix A

**Table A1:** Imputation results by reason.

Reference	#PJI	#	Baseline	Culture-	Polymicrobial	Single	Test	Adjusted	Adjusted	Adjusted	
	cases	*Candida*	fPJI	negative	adjust.	fungal	method	total	#PJI	proportion	
		reported	incidence	adjust.		culture	adjust.	*Candida*	cases	*Candida*	
						adjust.					
Lyu et al. (2024)	219	13	5.9 %	0				13	219	5.9 %	Min.
Lyu et al. (2024)	219	13	5.9 %	0				13	219	5.9 %	Max.
Baecker et al. (2021)	623	20	3.2 %	0				20	623	3.2 %	Min.
Baecker et al. (2021)	623	20	3.2 %	8				28	700	4.0 %	Max.
Lee et al. (2024)	181	13	7.2 %	4				17	181	9.4 %	Min.
Lee et al. (2024)	181	13	7.2 %	4				17	181	9.4 %	Max.
Lin et al. (2025)	271	16	5.9 %	1				17	271	6.3 %	Min.
Lin et al. (2025)	271	16	5.9 %	1				17	271	6.3 %	Max.
Morreel et al. (2025)	187	2	1.1 %	2				4	210	1.9 %	Min.
Morreel et al. (2025)	187	2	1.1 %	2				4	210	1.9 %	Max.
Tai et al. (2022b)	1162	79	3.4 %	6				85	1162	7.3 %	Min.
Tai et al. (2022b)	1162	79	3.4 %	6				85	1162	7.3 %	Max.
Wu et al. (2025)	201	4	2.0 %	2				10	201	5.0 %	Min.
Wu et al. (2025)	201	4	2.0 %	2				10	201	5.0 %	Max.
Aunon et al. (2025)	499	12	2.4 %	0		0		12	499	2.4 %	Min.
Aunon et al. (2025)	499	12	2.4 %	6		4		22	566	3.9 %	Max.
Froschen et al. (2022)	432	17	3.9 %	0		0		17	432	3.9 %	Min.
Froschen et al. (2022)	432	17	3.9 %	5		3		25	488	5.1 %	Max.
Jiang et al. (2025)	255	7	2.7 %	3		0		10	255	3.9 %	Min.
Jiang et al. (2025)	255	7	2.7 %	3		2		12	257	4.7 %	Max.
Ull et al. (2020)	124	5	4.0 %	0		1		6	125	4.8 %	Min.
Ull et al. (2020)	124	5	4.0 %	0		1		6	126	4.8 %	Max.
Brown et al. (2018)	3525	49	0.9 %	0			0	49	3525	1.4 %	Min.
Brown et al. (2018)	3525	49	0.9 %	43			23	115	3959	2.9 %	Max.
Herndon et al. (2023)	3989	73	1.8 %	49			0	122	4481	2.7 %	Min.
Herndon et al. (2023)	3989	73	1.8 %	49			26	148	4481	3.3 %	Max.
Tsai et al. (2019)	294	10	3.4 %	8			0	18	294	6.1 %	Min.
Tsai et al. (2019)	294	10	3.4 %	8			2	20	294	6.8 %	Max.
Budin et al. (2025)	3645	47	1.3 %	45		0	0	92	4094	2.2 %	Min.
Budin et al. (2025)	3645	47	1.3 %	45		29	24	145	4122	3.5 %	Max.
Cao et al. (2025)	278	28	10.1 %	6		0	0	34	278	12.2 %	Min.
Cao et al. (2025)	278	28	10.1 %	6		2	2	38	280	13.6 %	Max.
Kuo et al. (2018)	1184	29	2.4 %	0		9	0	38	1193	3.2 %	Min.
Kuo et al. (2018)	1184	29	2.4 %	15		9	8	61	1339	4.6 %	Max.
Sidhu et al. (2019)	1189	22	1.9 %	0		0	0	22	1189	1.9 %	Min.
Sidhu et al. (2019)	1189	22	1.9 %	15		9	8	54	1345	4.0 %	Max.
Benito et al. (2016)	2524	30	1.2 %	24	84	0	0	138	2524	5.5 %	Min.
Benito et al. (2016)	2524	30	1.2 %	24	84	20	17	175	2545	6.9 %	Max.
Ergin et al. (2024)	2469	49	2.0 %	30	161	0	0	240	2773	8.7 %	Min.
Ergin et al. (2024)	2469	49	2.0 %	30	161	19	16	275	2792	9.8 %	Max.
Ergin et al. (2025)	4261	45	1.1 %	53	243	0	0	341	4786	7.1 %	Min.
Ergin et al. (2025)	4261	45	1.1 %	53	243	33	28	402	4819	8.3 %	Max.
Haraldsdottir et al. (2025)	293	6	2.0 %	3	8	0	0	17	293	5.8 %	Min.
Haraldsdottir et al. (2025)	293	6	2.0 %	3	8	2	2	21	294	7.1 %	Max.
Yang et al. (2024)	348	14	4.0 %	8	14	0	0	36	348	10.3 %	Min.
Yang et al. (2024)	348	14	4.0 %	8	14	3	2	41	351	11.7 %	Max.
Totals and grand mean (%)		590		290	510	73	79			5.07 %	
